# EDMR: An Enhanced Dynamic Multi-Hop Routing Protocol with a Novel Sleeping Mechanism for Wireless Sensor Networks

**DOI:** 10.3390/s25144510

**Published:** 2025-07-21

**Authors:** Emad Alnawafa, Mohammad Allaymoun

**Affiliations:** 1 Electrical Engineering Department, Faculty of Engineering Technology, Al-Balqa Applied University, Salt 19117, Jordan; 2 Administrative Science Department, College of Administrative and Financial Science, Gulf University, Sanad 26489, Bahrain; alkarak1@yahoo.com

**Keywords:** wireless sensor network, LEACH protocol, DMR protocol, clustering, energy depletion, network lifetime, stability, throughput

## Abstract

Numerous protocols have emerged to address the energy depletion problem in Wireless Sensor Networks (WSNs). Among these protocols, the Dynamic Multi-Hop Routing (DMR) protocol adopts a dynamic technique for routing data across the network. The use of the DMR protocol has shown promising results in reducing energy consumption, prolonging the network lifetime, and increasing throughput. To improve the performance of WSNs, this paper proposes the Enhanced Dynamic Multi-Hop Routing (EDMR) protocol as a modification of the DMR protocol. The EDMR protocol introduces an effective sleeping mechanism that selectively deactivates clusters that do not generate significantly updated data for a specific duration. This mechanism reduces redundant transmissions, thereby saving energy and prolonging the network lifetime. The EDMR protocol incorporates static and dynamic approaches to support two major categories of applications: monitoring and event-driven applications. The proposed protocol is evaluated against the DMR protocol, the Enhanced Dynamic Multi-Hop Technique (EMDHT-LEACH) protocol, and the Low-Energy Adaptive Clustering Hierarchy (LEACH) protocol. The simulation results demonstrate that the EDMR protocol mitigates energy depletion, extends the network lifetime, increases stability, and improves network throughput toward the Base Station (BS), while reducing packet redundancy compared with the other protocols.

## 1. Introduction

WSNs consist of a multitude of sensor nodes that collaborate to fulfill the purpose of the network. Typically, these sensor nodes are deployed either uniformly or randomly throughout the environment. In most scenarios, the primary purpose of a WSN is to collect environmental data or track specific targets within the monitored area. Accordingly, the nodes begin sensing the region and transmitting their data to the BS. The aggregated data at the BS are either processed locally or transmitted to an external entity such as an end user or a control center. As a result, many applications for WSNs have emerged in modern times, spanning various sectors, including industry, agriculture, healthcare, and the military [1,2]. WSNs have special characteristics that distinguish them from other wireless networks. Among these characteristics are limited energy resources, data storage, and computational capabilities. A sensor node is the fundamental unit of the network; in most applications, it faces various challenges that affect the network’s performance. Energy depletion in the sensor nodes is one of these challenges; in most cases, it is not possible to replenish energy from another source, especially when these nodes are deployed in extensive and harsh environments. Most of the sensor nodes’ energy is consumed during data transmission to the BS [3]. Hence, much research has focused on minimizing the energy required for data transmission, which in turn prolongs the network’s lifetime and increases its efficiency. Several routing protocols have been introduced to manage data transmission across the network area—specifically, the transmissions from the sensor nodes to the BS.

Generally, routing protocols are categorized into three groups based on the wireless network architecture: flat routing protocols, location-based routing protocols, and hierarchical routing protocols [4]. Each category has a specific technique for forwarding data from sensor nodes to the BS. Among these, the hierarchical category is the most common. It provides a routing mechanism based on grouping all sensor nodes. An example of a protocol in this category is the LEACH protocol [5]. The main idea of the LEACH protocol is based on distributing all the sensor nodes into clusters. Each cluster comprises a number of nodes responsible for gathering data from the environment, which are then transmitted to an aggregator sensor node called the Cluster Head (CH). Afterwards, all the CHs send the collected data to the BS. The LEACH protocol divides the lifespan of the network into rounds. Each round is split into two phases: the setup phase and the steady-state phase. The setup phase is dedicated to forming the clusters and electing the CHs. At the start of each round, all sensor nodes select a random number between 0 and 1. This random value is compared with a threshold value, *T*(*n*). If it is lower than *T*(*n*), the sensor node is selected as the CH for that round. *T*(*n*) is calculated according to Equation (1) [5]:

(1) $$T\left(n\right)=\left\{\begin{array}{c} \frac{p}{1-p(r\;mod\frac{1}{p})}\;\;\;\;\;\;\;\;\;\;\;\;\;if\;n\in G\;\;\;\;\\ \;\;\;\;\;0\;\;\;\;\;\;\;\;\;\;\;\;\;\;\;\;\;otherwise\;\;\;\;\; \end{array}\right.$$

In this context, the parameter *p* represents the percentage of CHs in each round, the parameter *r* denotes the index of the current round, and the parameter *G* identifies the group of sensor nodes that have not been elected as CHs in the previous 1/*p* rounds.

Subsequently, each CH begins forming its cluster by broadcasting announcement messages to the remaining nodes, which were not selected as CHs in the current round and are referred to as normal nodes (Ns). In response, each N determines the appropriate CH to join for the current round. Usually, the selection of a suitable CH depends on the Received Signal Strength Indicator (RSSI). Consequently, each N transmits a Join-Request (JOIN-REQ) message to the CH with the highest RSSI and waits for an acknowledgment. To coordinate data transmissions within the cluster, the CH generates a Time-Division Multiple Access (TDMA) schedule that assigns a specific time slot to each node. Thus, each node can transmit during its assigned slot. The steady-state phase follows the setup phase, during which the Ns collect data from the environment and then transmit the data to their CH. The collected data are compressed and directly sent by the CH to the BS. To eliminate the impact of interference that may occur between clusters, a Code-Division Multiple Access (CDMA) code can be employed [6].

The LEACH protocol offers various advantages for WSNs such as enhancing the network’s energy efficiency by dividing all the network nodes into clusters. It assumes that the CHs are rotated every round, which in turn ensures balance among the nodes and extends the network lifetime. However, the LEACH protocol faces several challenges that lead to increased energy consumption. One such challenge is the extensive deployment area, where CHs require considerable energy to transmit the aggregated data to the BS. Furthermore, the LEACH protocol assumes that all the network nodes must remain in the active state throughout the network’s lifetime. This assumption increases the amount of redundant data transmitted within the network, thereby increasing energy consumption and decreasing network performance [7]. Therefore, several protocols have been presented to overcome these challenges. The DMR protocol is considered one of them [8]. It provides a dynamic approach for data transmission among all the network nodes, taking into account the remaining energy of the nodes, their locations inside and outside the clusters, and their distances to the CHs and the BS. Additionally, it introduces new approaches for cluster formation, electing the CHs, and determining the maximum number of normal nodes in each cluster. The results of using this protocol revealed a significant improvement in energy consumption, which positively impacted the network’s performance compared to the LEACH protocol and several previous protocols in this domain.

On the other hand, the DMR protocol assumes that all sensor nodes, whether CHs or Ns, are active and continuously transmit their data to the CHs, which subsequently forward the collected data toward the BS. A considerable portion of the data is redundant, or no substantial update has occurred in the previous data. In this case, it is clear that a notable loss of energy is wasted unnecessarily. Due to the aforementioned reasons, the EDMR protocol is proposed in this paper. The main contributions of this paper are summarized as follows:

(1) Propose the EDMR protocol as an enhanced version of the DMR protocol to reduce energy dissipation and prolong the network’s lifetime;
(2) Introduce a novel sleeping mechanism to selectively deactivate clusters that have not transmitted new or significantly updated data to the BS. Unlike many previous sleep-based protocols that maintain fixed sleeping approaches at the node or cluster level throughout each round, the proposed protocol provides two alternative sleeping scenarios based on the WSN’s demands:
  - The static sleep scenario is designed for monitoring applications, where the sensed data typically do not change rapidly. Consequently, maximum energy savings can be achieved while preserving the network’s performance and intended functionality;
  - The dynamic sleep scenario departs from static assumptions by allowing sleep/wake states to be adjusted within the same round. Accordingly, the cluster nodes update their status in response to real-time events occurring within the network. This intra-round adaptability enables efficient energy conservation while maintaining the network’s coverage and performance;
(3) Utilize the INs distributed across the network, including those located near sleeping clusters, to collect and forward data to the BS. These INs also provide valuable feedback that supports future sleep decisions. By combining the dual-sleep mode design with real-time operational awareness, the EDMR protocol offers a more adaptable sleep management strategy than traditional protocols, which are typically based on fixed or non-responsive mechanisms;
(4) Integrate the proposed sleeping mechanism with the dynamic routing technique to ensure consistent data flow and minimize energy usage.

This paper is organized as follows: Section 2 introduces related data routing protocols and sleeping techniques proposed for WSNs. Section 3 presents a detailed explanation of all phases of the DMR protocol. Section 4 is dedicated to the new modification techniques proposed by the EDMR protocol. Section 5 discusses the results obtained from applying the EDMR protocol. Lastly, Section 6 concludes the paper.

## 2. Related Work

Many protocols have been proposed to boost the performance of WSNs by improving data transmission within them. The majority of these protocols have achieved promising results by using multi-hop techniques to route data across the WSN. These techniques vary between static and dynamic approaches. In the literature, several multi-hop routing protocols that use a static approach have been proposed such as the Multi-Hop based on the LEACH (MH-LEACH) protocol [9]. It introduces two phases for selecting efficient paths from CHs to the BS: an initialization phase and a steady-state phase. The initialization phase is responsible for forming the clusters and follows the same procedure as the original LEACH. Therefore, the CHs broadcast announcement messages to all non-CHs, inviting them to join their clusters. Then, the non-CH nodes select the most suitable CH based on RSSI. Once the cluster formation is complete, each CH transmits its routing table to the BS, which in turn carefully checks the routing table. If the BS identifies intermediate CHs between the source CH and the BS, it rearranges the routing table accordingly and sends it back to the source CH. The design of the MH-LEACH protocol contributes to reducing the energy required for transmitting the aggregated data to the BS, thereby extending the network lifetime. Despite its ability to curb transmission energy, the MH-LEACH protocol uses a fixed route from the CH to the BS throughout each round. Furthermore, it does not support any sleep-scheduling mechanism, leaving all nodes permanently active, which leads to continuous energy drain.

To address the energy imbalance issue in the original LEACH, the LEACH-TL protocol was introduced in [10]. Instead of one CH, LEACH-TL assumes that each cluster should have two CHs to mitigate energy depletion, which may occur if the selected CH has low residual energy. Accordingly, one of them, referred to as the secondary CH, is responsible for gathering data from the cluster’s member nodes, while the second, known as the main CH, is used to transmit the aggregated data to the BS. If the residual energy of the main CH decreases to less than half, the secondary CH takes responsibility for transmitting the data to the BS. In certain cases, the aggregated data of the main CH can be transmitted to the BS via another main CH. LEACH-TL prolongs the overall network lifetime by improving energy balancing within the clusters. However, no sleep-scheduling mechanism is employed; the protocol relies on static routes for data delivery to the BS.

Alternatively, various protocols have adopted dynamic multi-hop routing approaches in WSNs. For example, the EDMHT-LEACH protocol proposed in [11], is considered an enhanced version of the DMHT-LEACH protocol. It introduces several modifications, including adjustments to the equation used for CH selection and to the number of member nodes in each cluster. Furthermore, it defines a new type of node called an Independent Node (IN), which is not affiliated with any specific cluster. INs also participate in routing the CH data to the BS. The EDMHT-LEACH protocol achieves a better energy balance by adjusting the cluster size and controlling the number of nodes within each cluster. The use of INs reduces the load on CHs and optimizes the overall network’s performance. However, the EDMHT-LEACH protocol does not employ any sleep-scheduling mechanism, leading to an increase in unnecessary energy dissipation.

From another perspective, several protocols have introduced active/sleep mechanisms for the network nodes. The authors in [12] employed the K-means algorithm to modify the LEACH protocol. The algorithm contributed to the selection of the CHs. Furthermore, they proposed an active/sleep scheduling mechanism for the cluster member nodes. Accordingly, nodes in sleep mode turn off their transmitter and receiver modules to minimize energy consumption, while active nodes continue to operate normally. Thus, the K-means algorithm improves the CH selection process by considering spatial proximity, while energy consumption is minimized through the use of the sleep mechanism. However, the protocol overlooks the residual energy of the nodes during CH selection. Moreover, it relies on single-hop communication between the CH and the BS. Its sleep mechanism is managed locally.

The Energy-Efficient Sleep–Awake Aware (EESAA) protocol was proposed in [13]. It provides a pairing-based strategy to enhance energy efficiency in homogeneous WSNs. During the setup phase, the BS groups neighboring sensor nodes into pairs. In each round, one node from each pair becomes active and is responsible for sensing and communication, while the other remains in sleep mode. The EESAA protocol selects the CHs using a probabilistic model that considers the residual energy of the nodes. Once selected, each CH directly transmits the collected data to the BS through single-hop communication, without relying on intermediate nodes. The EESAA protocol boosts the network’s stability and reduces energy consumption by minimizing redundant transmissions. It does not support multi-hop communication. Moreover, sleep-scheduling decisions are entirely made at the pair level, without any network-wide coordination.

To reduce energy consumption and prolong the lifetime of the WSN, the Sleep–awake Energy-Efficient Distributed (SEED) protocol was developed in [14]. It divides the network area into three regions based on the distance from the BS. Within each region, sensor nodes with similar sensing responsibilities are grouped into sub-clusters. The sleep-scheduling mechanism used by the SEED protocol relies on local decisions. In each round, only one sensor node in each sub-cluster remains active, while the others enter sleep mode. The SEED protocol employs a distributed approach to select CHs, taking into account each node’s remaining energy and the number of times it has previously served as a CH. It utilizes single-hop communication to forward the collected data from the CHs to the BS. Simulation results have revealed that the SEED protocol reduces energy dissipation and extends the lifetime of the WSN.

Threshold-based LEACH with Sleep/Awake Scheduling (T-LEACHSAS) was proposed in [15] as an enhanced version of the T-LEACH protocol. The T-LEACHSAS protocol integrates a threshold-based cluster-head selection strategy with a scheduled sleep–awake mechanism. The CH selection process is based on the residual energy of each node and a predefined energy threshold. Accordingly, a CH continues to operate across multiple rounds as long as its residual energy remains above the threshold. Each CH utilizes a TDMA-based schedule, where each non-CH becomes active during its assigned time slot and remains in sleep mode during the remaining time. A single-hop communication model is used to transmit the aggregated data from the CH to the BS. The T-LEACHSAS protocol helps to conserve energy and prolong the lifetime of the WSN.

To mitigate the rapid energy depletion of hotspot nodes and enhance overall energy efficiency, the Interval Type-2 Fuzzy Unequal Clustering and Sleep Scheduling (IT2FUSS) protocol was proposed in [16]. It employs an Interval Type-2 Fuzzy Inference System (IT2-FLS) to enable each node to make local decisions based on multiple parameters, including its residual energy, its distance to the BS, and its neighborhood density. The IT2FUSS protocol dynamically adjusts the cluster sizes according to the distance of each node to the BS, assigning smaller radii to nodes located closer to the BS. In each round, IT2FUSS enables a node to enter sleep mode unless it is considered critical by the fuzzy logic. It is worth mentioning that the IT2FUSS protocol leverages single-hop communication to transmit the CHs’ data to the BS. Simulation results showed that the IT2FUSS protocol effectively balances energy consumption and prolongs the overall network lifetime.

The Energy and Throughput Aware Adequate Routing (ETAAR) protocol was introduced in [17]. It offers an approach to improve both data transmission and energy efficiency in WSNs. The ETAAR protocol selects the CHs based on multiple factors such as their distance to the BS, residual energy, throughput, and delay. A TDMA-based sleep-scheduling mechanism is applied to reduce energy consumption. Additionally, the protocol forwards data to the BS using a multi-hop technique.

The Dynamic Energy-Aware MAC Extension (DE-MAC Extension) protocol was developed in [18]. It aims to reduce unnecessary transmissions and prolong the WSN’s lifetime. During each round, sensor nodes send their residual energy to their respective CHs, which then allocate a specific number of TDMA time slots to each node. Nodes with higher energy are granted more transmission slots, whereas nodes with lower energy receive fewer slots and remain in sleep mode for longer periods. The DE-MAC Extension protocol utilizes single-hop communication to transmit the aggregated data from the CHs to the BS. The Reinforcement of MIS-Based Sleep Scheduling (RMIS) protocol introduces a hybrid approach in [19]. This approach incorporates the Maximum Independent Set (MIS) theory with reinforcement learning to determine effective sleep scheduling. The protocol divides the network into clusters; within each cluster, a subset of nodes is selected to remain active. The selection of the nodes is based on their energy levels, historical activity, and neighborhood density. The RMIS protocol significantly minimizes energy dissipation by reducing the number of active nodes while preserving the network’s coverage. However, it employs a uniform sleep strategy and does not consider the specific requirements of WSN applications.

The Reinforcement Learning-based Cooperative Event-driven (RL-Coop Event) protocol was proposed in [20]. It provides an event-driven sleep scheduling strategy designed for large-scale clustered WSNs. In this protocol, clusters are dynamically formed using the K-means algorithm, typically after the detection of an event. To determine the active nodes within each cluster, the Deep Deterministic Policy Gradient (DDPG) model is employed. The selected active nodes are responsible for sensing and processing the event, while the remaining nodes are deactivated. By only activating the necessary nodes, the RL-Coop Event protocol reduces energy dissipation and extends the network’s lifetime. Nonetheless, the RL-Coop Event protocol lacks multi-hop communication support. In addition, it does not provide adaptability based on the application requirements.

Table 1 presents a comparative summary of the protocols discussed in this section, including the proposed EDMR protocol. The comparison is based on their sleep-scheduling mechanisms; the control level of sleep decisions, routing methods, application-adapted sleep strategies; and the use of INs.

## 3. Dynamic Multi-Hop Routing (DMR) Technique

The DMR protocol dynamically switches between available routes through the WSN. The results of applying the DMR protocol have revealed that distributing the load across different sensor nodes along the route can improve the WSN’s performance, extend its lifetime, and increase its overall stability. The protocol divides the network’s lifetime into successive rounds, each comprising four phases: initialization, announcement, table preparation, and routing. The DMR protocol makes several assumptions about the WSN architecture used within it, as follows: the sensor nodes are deployed randomly across the network area and are initially charged with the same amount of energy; the network includes a BS; and the distances of the CHs are measured relative to it. The first section introduces the energy dissipation model used by the DMR protocol, followed by an explanation of the protocol’s phases.

### 3.1. Energy Dissipation Model

The DMR protocol employs the energy dissipation model presented in [6]. This model is used to compute the energy required to transmit or receive a K-bit packet. Additionally, this model is applied to calculate the energy consumed by control packets. Here, Equations (2) and (3) are used for transmission and reception, respectively:

(2) $$E_{Tx}(k,\;d)=\left\{\begin{array}{c} E_{elec}\times k+\varepsilon_{fs}\times k\times d^{2},\;\;\;\;\;d<d_{o}\\ E_{elec}\times k+\varepsilon_{mp}\times k\times d^{4},\;\;\;\;\;d\geq d_{o} \end{array}\right.$$

(3) $$E_{Rx}\left(k\right)=E_{elec}\times k$$

In this context, *E_elec_* denotes the amount of energy required per bit for handling data transmitted or received across the WSN. Both the $\varepsilon_{fs}$ and $\varepsilon_{mp}$ parameters correspond to the free-space propagation model and the two-ray propagation model, respectively. It is worth mentioning that $\varepsilon_{fs}$ is used when the transmission distance is less than *d_o_*, while $\varepsilon_{mp}$ is applied otherwise. The threshold distance *d_o_* is given by Equation (4) [6]:

(4) $$d_{o}=\sqrt{\varepsilon_{fs}/\varepsilon_{mp}}$$

### 3.2. Phases of the DMR Protocol

#### 3.2.1. The Initialization Phase

As mentioned above, the round starts with the initialization phase, which is responsible for creating the network clusters. First, each node generates a random number in the range between 0 and 1. This value is then compared with a threshold value *T*′(*i*). If it is less than *T*′(*i*), the node is elected as the CH for the current round. Otherwise, the node remains as a normal node and attempts to join a nearby cluster. The value of *T*′(*i*), used in the DMR protocol, was introduced in [21] and is computed using Equation (5). It is considered a modified version of Equation (1), as follows:

(5) $$T^{\prime }\left(i\right)=\left\{\begin{array}{c} max\left(\frac{p}{1-p\left(r\;mod\frac{1}{p}\right)}\times \frac{E_{residual}}{E_{max}},T_{min}\right)\;\;\;\forall_{i}\in G\\ 0\;\;\;\;\;\;\;\;\;\;\;\;\;\;\;\;\;\;\;\;\;\;\;\;\;\;\;\;\;\;\;\;\;\;\;\;\;\;\;\forall_{i}\notin G \end{array}\right.$$

Here, *E_residual_* denotes the remaining energy in a node after it has operated for a number of rounds. *E_max_* indicates the maximum energy level assigned to a node before it begins its task. *T_min_* indicates the minimum value of the threshold when the *E_residual_* of the node decreases to its minimum level. Once the CH-election process is completed, each CH becomes capable of forming clusters. To maintain balanced energy dissipation among the clusters, the DMR protocol determines the member nodes in each cluster. Thus, each CH manages a limited number of nodes within its cluster. For a network with *J* nodes, the number of *CHs* is estimated using Equation (6). Accordingly, the number of member nodes $N_{O}$ is given in Equation (7) and should not exceed this value [11]:

(6) $$Number\;of\;the\;{CH}_{s}=\sum_{n=1}^{J}p\times 1=J\times p$$

(7) $$N_{O}=\frac{J}{Number\;of\;the\;{CH}_{S}}$$

The cluster formation process begins with the announcements. Each CH announces to the surrounding ordinary nodes to join its cluster in the current round. The announcement message includes the CH identification (CH ID) and its geographical coordinates. In response, the Ns receiving these announcements calculate their distances to these CHs. The computed distances are recorded in their selection tables (STs) and sorted in descending order. Accordingly, each N sends its JOIN-REQ message to the closest CH, provided that the distance is less than d_o_, whereas the other distances are disregarded. Each CH checks its TDMA schedule for an available slot to serve the JOIN-REQ. If a free slot is found, it is assigned to the requesting node and an acceptance message is sent. Otherwise, a refusal message is transmitted. In that case, the node proceeds to the next option in the ST and sends a JOIN-REQ to the corresponding CH, and so forth. As a result of these cases, most of the nodes belong to their clusters, while some nodes fail to join any cluster. Hence, the DMR protocol considers the nodes that do not participate in any cluster as INs. These INs perform tasks similar to those accomplished by the CHs. Similar to the CHs, they contribute towards sensing data and routing these data directly to the BS or via other CHs or INs across multiple levels. They also assist CHs in routing their data to the BS.

The DMR protocol divides the entire network architecture into levels with respect to the BS. It assigns a length of d_o_/2 to each level. Hence, the first level includes all the CHs and INs located at distances less than d_o_/2, while the boundary of the second level lies at distances greater than, or equal to, d_o_/2 and less than d_o_, and so on. Additionally, the DMR protocol assumes that the network clusters are composed of two levels. The first level contains all Ns located at distances less than d_o_/2, whereas the second level contains the Ns located at distances greater than or equal to d_o_/2. Based on this distribution, the DMR protocol ensures that most data transmissions occur within a distance less than d_o_/2. This leveling approach, whether at the inter-cluster or intra-cluster level, positively affects the amount of energy dissipation and extends the network’s lifetime.

**Figure 1:**
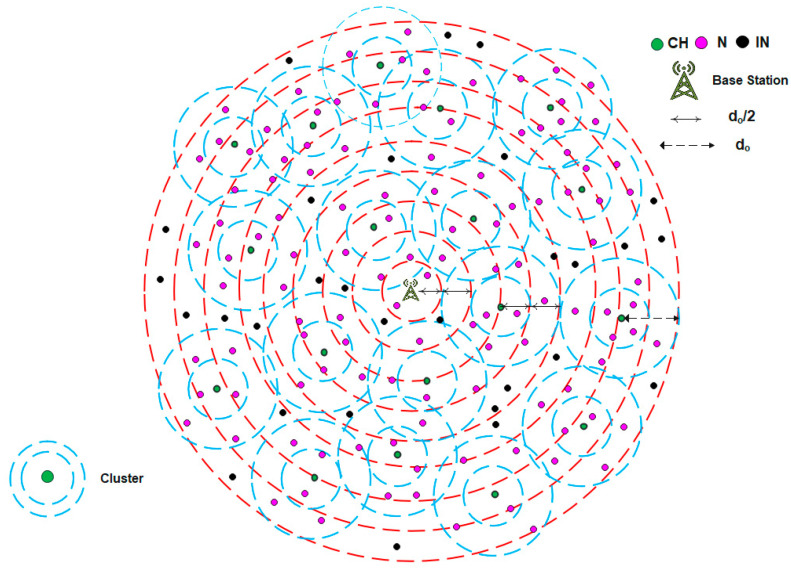
The overall architecture of WSN using the DMR protocol.

Figure 1 illustrates the overall architecture of the WSN using the DMR protocol. As described earlier, the entire network is divided into concentric levels, each with a length of d_o_/2. Consequently, the CHs and INs are distributed across these levels based on their respective distances from the BS. Furthermore, each cluster is internally divided into two levels, each having a length of d_o_/2. Accordingly, the Ns within each cluster are assigned to levels according to their distances from the corresponding CH.

#### 3.2.2. The Announcement Phase

In this phase, the DMR protocol assumes that all types of network nodes (CHs, INs, and Ns) periodically broadcast announcement messages. These messages declare the network nodes to each other. The DMR protocol utilizes two types of announcement messages. The first type is broadcast at the beginning of each round. Accordingly, Ns broadcast announcement messages to one another within each cluster. Typically, each announcement message includes the node’s ID and its corresponding cluster ID, coordinates, level, and residual energy. All CHs and INs also exchange their own announcement messages, which include their IDs, geographical coordinates, levels, and residual energy. The second type is an updated message, which is usually broadcast during the round. The updated message broadcast by an N includes its ID, corresponding cluster ID, and residual energy, while the one broadcast by the CHs and INs contains their ID, level, and residual energy.

#### 3.2.3. The Table Preparation Phase

For each network node—whether a CH, IN, or N—the previous phase provides a clear view of the surrounding nodes. Based on the announcement messages received, the nodes begin developing their routing tables. Consequently, the Ns located at the second level of the clusters dispatch JOIN-REQ messages to the Ns at the first level. The N receiving this request identifies a time slot in its TDMA schedule; if a free slot is available, it sends an acceptance message to the requester. Otherwise, the request is ignored. The requester updates its routing table (RT) based on the Ns that have sent their approvals. It is worth noting that a cost function is used to arrange the table’s entries. Moreover, the CHs and INs positioned at the second and upper levels attempt to construct their RTs by transmitting JOIN-REQ messages to the CHs and INs at the lower levels. The CHs and INs that send acceptance messages become candidates for the requester, which then arranges them in its routing table.

#### 3.2.4. The Routing Phase of the DMR Protocol

The DMR protocol assumes that the completion of the previous three phases ensures that all network nodes have their RTs. Therefore, they become capable of sensing data from the environment and conveying these data to the BS through other network nodes. In this phase, a dynamic technique is proposed for routing the sensed data intra-cluster and the aggregated data inter-cluster in order to deliver these to the BS. The following is an explanation of the proposed approach.

- Intra-cluster Routing

As previously highlighted, the nodes of each cluster are distributed into two levels around the CH. The Ns at the first level directly transmit the collected data to their corresponding CH. Each N located at the second level utilizes its RT to forward the data to the CH. The arrangement of entries within the RT depends on a cost function proposed for this purpose. The first component of this function corresponds to the relative position of the next-hop node (*NHN*) with respect to the *CH*, while the second component reflects the energy ratio of the *NHN*. Thus, the cost value for each *NHN* is calculated using Equation (8) [8]:

(8) $$C_{NHN}\left(i\right)=C_{NHN-CH}\left(i\right)+\alpha \cdot C_{NHNE}\left(i\right)$$

At this point, $C_{NHN-CH}$ and $C_{NHNE}$ denote the distance and energy cost components, respectively. The variable *i* indicates the nodes selected to calculate their costs. Finally, the parameter α represents the relative weight of the energy cost component within the cost function. Using Equation (9), the value of $C_{NHN-CH}$ is computed as follows [8]:

(9) $$C_{NHN-CH}\left(i\right)=\frac{{D_{i}}^{2}}{{SN}_{D-CH\;}-{\;NHN}_{iD-CH}}$$

In this context, $D_{i}$ denotes the distance separating the source node (*SN*) and the *NHN*. ${SN}_{D-CH}$ symbolizes the distance from the *SN* to the *CH*, while ${NHN}_{iD-CH}$ represents the distance between the *NHN* and the *CH*. Equation (10) is used to compute the energy ratio of the *NHN*:

(10) $$Energy\;ratio=\frac{{\;RE}_{NHN}}{{\;IE}_{NHN}}$$

**Figure 2:**
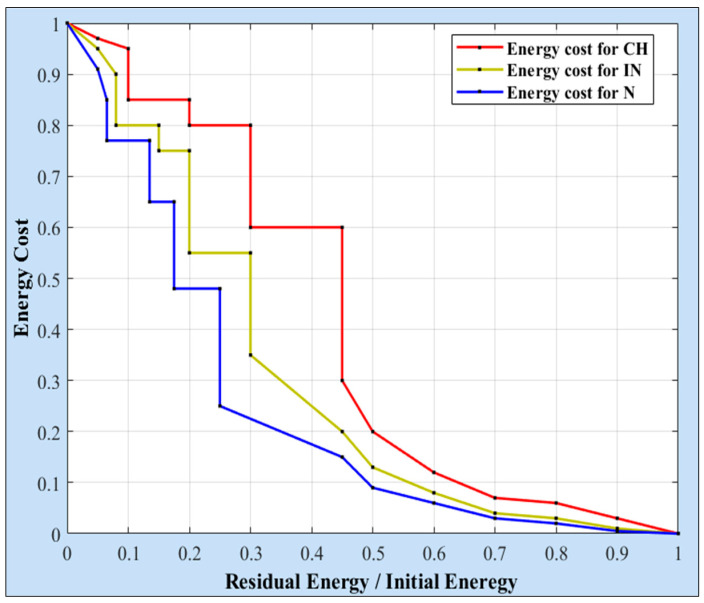
Example of energy cost values for CH, IN, and N nodes.

In this formulation, *RE_NHN_* refers to the residual energy of the *NHN*, while *IE_NHN_* indicates its initial energy. For each energy ratio, there is a corresponding cost value. Hence, lower cost values are assigned to higher energy ratios, and vice versa. Figure 2 illustrates an example of this assumption. Specifically, lower cost values are allocated to Ns whose residual energy exceeds 50% of their initial energy, whereas higher cost values are assigned to the others.

Thus, each SN computes the cost of all possible routes and updates its RT in ascending order, from the lowest cost to the highest. Undoubtedly, the SN selects the route with the lowest cost and transmits its data through it to the CH. During the round, the Ns broadcast updated messages. Therefore, the cost values of the routes may change, leading to a rearrangement of the entries within the table. Based on the new arrangement, the SN selects the new lowest-cost route for transmission, and so on. It should be noted that the energy cost component,$\;C_{NHNE}$, in Equation (8) has relatively low values (ranging between 0 and 1) compared to the distance cost. For this reason, α is added to amplify its impact within the cost function. Assigning a small value to α (e.g., 1–10) results in a negligible influence on the energy cost, whereas a large value (e.g., 100) ensures a more balanced contribution from both energy and distance components;

- Inter-cluster Routing

The aggregated data from the CHs, as well as the data from the INs, are also transmitted across the network levels to the BS. Consequently, the RTs of all the CHs and INs in the second and upper levels are constructed. The CHs and INs that receive acceptance responses to their JOIN-REQ messages calculate the cost value for each route. All routes are sorted in descending order within the RT; the lowest cost route is selected first for data transmission. The cost value of each route is computed in the same manner as in the intra-cluster case. Equation (11) is used to determine the value of the route cost: the first term of this equation corresponds to the positional cost of the next-hop node (NHI), whether it is a CH or an IN, with respect to the BS, while the second term refers to the energy cost of the NHI [8]:

(11) $$C_{NHI}\left(n\right)=C_{NHI-BS}\left(n\right)+\alpha \cdot C_{NHIE}\left(n\right)$$

Here, $\;C_{NHI-BS}$ represents the distance cost of the *NHI* relative to the *BS*; $C_{NHIE}$ indicates the cost related to the remaining energy of the *NHI*. The variable *n* refers to the set of next-hop nodes, whether these are the *CH* or the IN. Equation (12) computes the $C_{NHI-BS}$ as follows [8]:

(12) $$C_{NHI-BS}\left(n\right)=\frac{{D_{n}}^{2}}{{SNI}_{D-BS\;}-{\;NHI}_{nD-BS}}$$

In this formulation, *D* refers to the distance separating the source node *SNI*, whether a CH or an IN, from the *NHI*. ${SNI}_{D-BS}$ represents the distance between the *SNI* and the BS, whereas the ${NHI}_{nD-BS}$ denotes the distance between the *NHI* and the *BS*. The second component of the cost function assigns an energy cost to each remaining energy ratio. Figure 2 illustrates an example of the energy cost values assigned to the CHs and the INs. As shown, lower cost values are given to nodes with higher energy ratios, especially those whose energy ratios exceed 50%. To reduce the burden on the CHs, the proposed approach allocates slightly lower cost values to the INs, thereby enhancing their role in forwarding data across the levels.

Based on the cost values, all CHs and INs arrange their available routes in ascending order. The lowest-cost route is initially selected to transmit data to a CH or IN at a lower level. Based on the updated messages received from the CHs or INs in the lower levels, the SNI rearranges the entries in its RT. Consequently, a new route can be dynamically selected to replace the initially chosen one.

## 4. Enhancing the Dynamic Multi-Hop Routing (EDMR) Protocol

**Figure 3:**
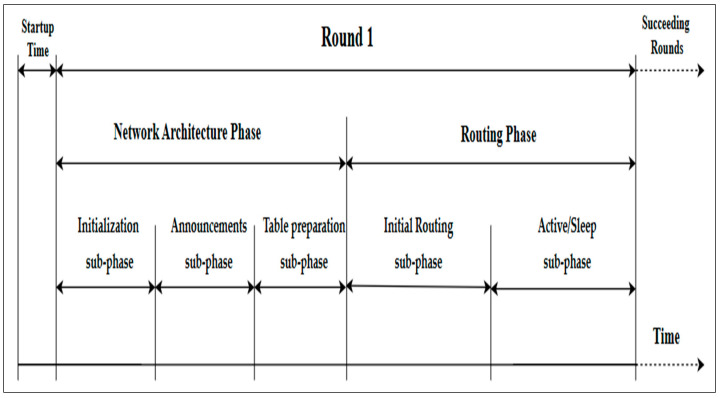
The phases of the EDMR protocol.

This paper presents new modifications to the DMR protocol. It aims to minimize the total energy consumption while preserving the WSN’s performance, stability, and throughput. For this reason, it proposes a new approach that retains the network architecture introduced in the DMR protocol; however, this approach makes adjustments to the number of clusters that should remain active for transmitting data toward the BS. Thus, the proposed protocol incorporates a sleep/wake mechanism to reduce the total energy consumption of the WSN, which in turn extends the network lifetime and improves the network throughput. It also considers the types of applications that use the WSN. The EDMR protocol divides the network lifetime into rounds. Each round includes two general phases: a network architecture phase and a data-routing phase, each consisting of a number of sub-phases, as shown in Figure 3. The following section provides explanations of the protocol’s phases.

### 4.1. The Network Architecture Phase

**Figure 4:**
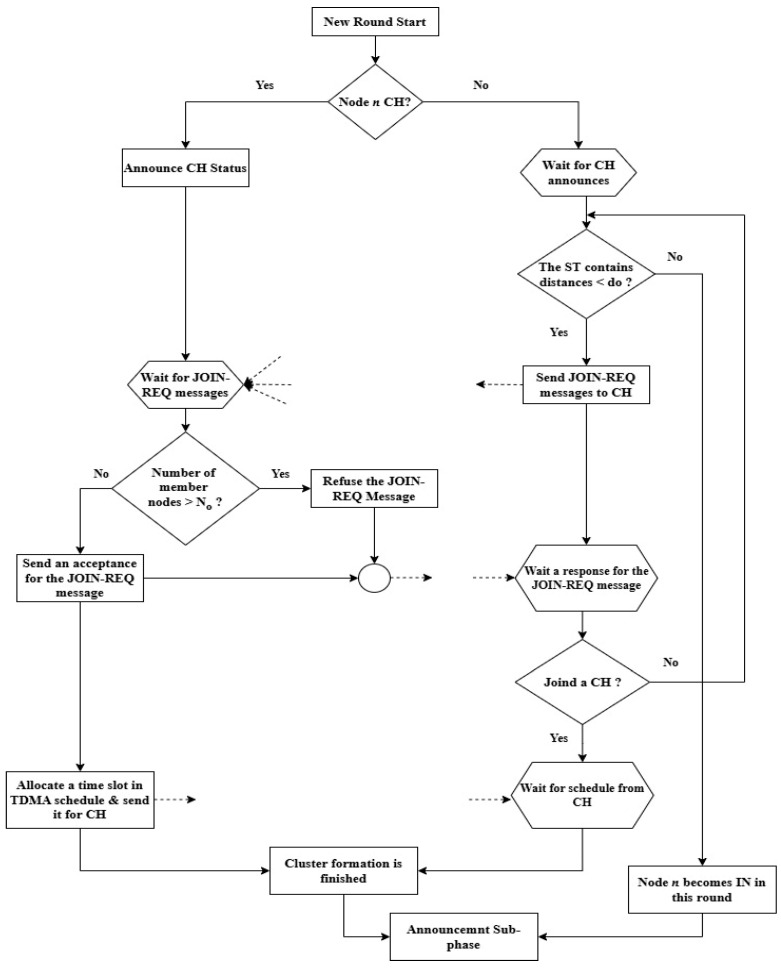
The initialization sub-phase of the EDMR protocol.

**Figure 5:**
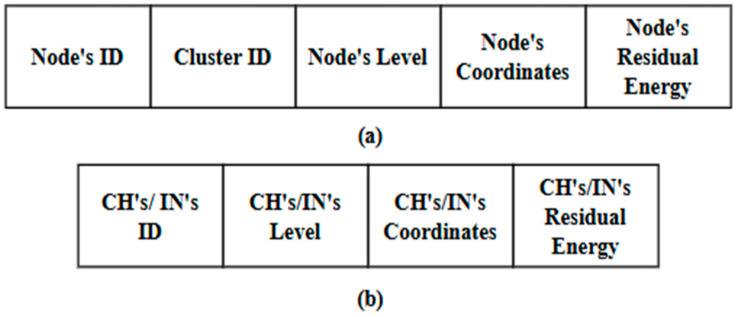
Components of the announcement message for: (**a**) Ns; and (**b**) CHs/INs.

**Figure 6:**
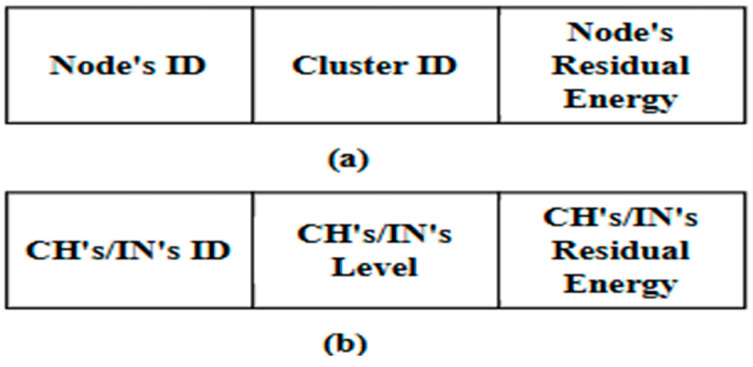
Components of the updated message for: (**a**) Ns; and (**b**) CHs/INs.

This phase is responsible for organizing all deployed sensor nodes into clusters. It includes three sub-phases: initialization, announcement, and table preparation. It is worth noting that the network architecture phase of the EDMR protocol builds upon the DMR phases previously described in Section 3.2.1, Section 3.2.2 and Section 3.2.3. For clarity, the flowchart shown in Figure 4 illustrates a summary of the initialization sub-phase. Figure 5 presents the announcement messages used by the Ns, CHs, and INs to create their RTs at the beginning of the round, while Figure 6 shows the updated messages used by them to update their RTs during the round. Finally, Algorithm 1 outlines the table preparation sub-phase. The table generated in this sub-phase is employed in the routing phase.
**Algorithm 1**: Table Preparation Sub-Phase
**Definitions** N[i] ∈ the set of Ns located at distance ≥ d_o_/2 from the CH
N[j] ∈ the set of Ns located in the lower levels
CH[m]/IN[m] ∈ the set of CHs/INs located at distance ≥ d_o_/2 from the BS CH[n]/IN[n] ∈ the set of CHs/INs located at distance ≥ do/2 from the BS RT: Routing Table
Ann_Mess: Announcement message 
Join_Req: Join Request message Acc_Mess: Acceptance message1:begin2: for each (N[i])3:    if (received Ann_Mess from N[j]) then4:      send Join_Req to N[j];5:    end if6:  for each (N[j]) received Join_Req7:      if (has available in its TDMA table) then8:         assign a slot to N[i];9:         send *Acc_Mess* to N[i];10:      end if11:   end for12:   for each (Acc_Mess received from N[j])13:        compute cost (N[i], N[j]);14:        add (N[j], cost (N[i], N[j]));15:        sort RT[i] in ascending order;16:    end for 17: end for18: for each (CH[m]/IN[m])19:    if (received Ann_Mess from CH[n]/IN[n]) then20:        send Join_Req to CH[n]/IN[n];21:    end If22:  for each (CH[n]/IN[n])23:      if (has available in its TDMA table)24:        assign a slot to CH[m]/IN[m];25:        send Acc_Mess to CH[m]/IN[m];26:      end if27:  end for28:   for each (Acc_Mess from CH[n]/IN[n])29:        compute cost (CH[m]/IN[m], CH[n]/IN[n]);30:        add (CH[n]/IN[n], cost (CH[m]/IN[m], CH[n]/IN[n]));31:        sort RT[m] in ascending order;32:    end for33:end for

### 4.2. The Routing Phase of the EDMR Protocol

This phase is responsible for forwarding the sensed data to the BS, where a dynamic routing approach is used for data transmission both within the clusters (intra-cluster) and between the CHs and INs across the network levels toward the BS (inter-cluster). The routing phase is divided into two sub-phases: the initial routing sub-phase and the active/sleep sub-phase. It is important to emphasize that the types of applications using this protocol are considered. Hence, the active/sleep sub-phase determines which clusters should be deactivated and which should remain active and continue operating. The sub-phases of this phase are discussed in detail below.

#### 4.2.1. The Initial Routing Sub-Phase

As previously mentioned, the different types of nodes (CHs, INs, and Ns) are ready to start their operations by the end of the table preparation sub-phase. The Ns begin sensing the environment, collecting data, and transmitting it to their CHs (intra-cluster). Each cluster is divided into two levels. Thus, the Ns of the first level transmit their data directly to the CHs, while the second-level Ns utilize their RTs to choose the first entry and route their data to the CH through it. The data gathered by the CHs must be sent to the BS (inter-cluster). Therefore, the CHs and INs located in the first level promptly send the collected data toward the BS, while the data from the CHs and INs in the second level are forwarded to other CHs and INs located at lower levels. The RTs of the CHs and INs are also used to select the first entry. Algorithm 2 summarizes the essential steps performed in this sub-phase. As a result, the data during the initial phase flow smoothly from all network nodes toward the BS, which in turn analyzes the collected data and determines the next structure of the network by selecting which clusters must remain active and which should deactivate their operations. This mechanism is employed in the following phase.
**Algorithm 2**: Initial Routing Sub-Phase
**Definitions** N[i] ∈ the set of Ns located at distance ≥ d_o_/2 from the BS 
CH[m]/IN[m] ∈ the set of CHs/INs located at distance ≥ d_o_/2 from the BS
RT: Routing Table
NXN: Next hop node (N)
NIN: Next hop node (CH/IN)
D_N[i]-CH_: Distance from N[i] to its corresponding CH 
R_RT[i]-CH_: Set of available routes in RT for N[i] to reach CH D_CH[m]/IN[m]-BS_: Distance from CH[m]/IN[m] to BS R_RT[m]-BS_: Set of available routes in RT for CH[m]/IN[m] to reach BS1:begin2: for each (N[i])3:     if (D**_N[i]-CH_** < d_o_/2)4:       transmit sensed data directly to the CH;5:      else if (D_N[i]-CH_ < R_ST[i]-CH_)6:        transmit sensed data directly to the CH;7:         else8:          select NXN from RT[i];9:           transmit the sensed data to the CH through it;10:       end if11:  end for12: for each (CH[m]/IN[m])13:      if (D_CH[m]/IN[m]-BS_ < d_o_/2) 14:        transmit aggregated data directly to the BS;15:         else If (D_CH[m]/IN[m]-BS_ < R_RT[m]-BS_)16:           transmit the aggregated data directly to the BS;17:            else18:             select NIN from RT[m];19:             transmit aggregated data to the BS through it;20:       end if21: end for

#### 4.2.2. The Active/Sleep Sub-Phase

It should be noted that the proposed protocol considers two general types of applications: monitoring applications (in which the sensed data remain relatively stable over time) [22], and event-driven applications (in which the sensed data change over time) [23]. Based on the application type, the EDMR protocol proposes two scenarios for the network structure and for routing the data within it during the rest of the current round. The first scenario is dedicated to monitoring applications. Here, the clusters chosen to be deactivated in this round remain inactive until the end of the round, whereas the second scenario is related to event-driven applications, in which the states of the clusters (active/deactivated) can be systematically changed throughout the current round. The following sections provide detailed explanations of the two scenarios.

##### Static Sleeping Scenario

WSNs used in this type of application typically collect data from the surrounding environment such as air temperature and soil humidity in agricultural applications. Therefore, the network’s BS is capable of analyzing the aggregated data from the initial routing phase and comparing it with a Threshold value (TH). Equation (13) demonstrates the relative difference (Δ) between the new data value (*V*_new_) sent to the BS and the previously stored data value (*V*_pre_):

(13) $$\Delta =\left|\frac{Vnew-Vpre}{Vpre}\right|$$

If Δ < TH, the change is considered insignificant. When the BS receives such data from a cluster, it selects that cluster to be deactivated for the rest of the round. It then sends a broadcast message that includes the CH ID and sets the mode bit to 1 (deactivated = 1, activated = 0) to inform the cluster to enter sleep mode for the remainder of the round. It is worth noting that the value of TH plays a key role in balancing energy consumption and maintaining acceptable coverage in the WSN. A lower TH value (e.g., 0.01) keeps more clusters active, resulting in increased data transmissions and faster energy depletion. Conversely, a higher TH value (e.g., 0.05) would reduce the energy consumption but may lead to the loss of valuable data. Therefore, selecting a moderate TH value (e.g., 0.03) strikes a balance between energy efficiency and network coverage. In this study, the TH was set to 0.03 as a design assumption. Upon receiving the broadcast message from the BS, the CH typically broadcasts a message instructing all cluster nodes to enter sleep mode, while the CH itself remains active throughout the remainder of the round. The main purpose of this procedure is to enable the CH to cooperate with other active CHs and INs in routing their data to the BS. Algorithm 3 presents the pseudocode for the static sleeping scenario.

At this point, the new structure of the network comprises the active clusters, the INs, and the CHs of the deactivated clusters. They utilize their RTs to forward data toward the BS. During the round, updated messages are sent within each cluster (intra-cluster) to update the entries of the selection tables of the Ns, so that the routes may dynamically change during the round toward their CHs. Similarly, updated messages are broadcast across the levels to update the RTs of the CHs and the INs, which may also dynamically change their routes toward the BS.
**Algorithm 3**: Static Sleeping Scenario
**Definitions**
CH[m] ∈ the set of CHs 
TH: Threshold value N: Normal node
*V_new_*: the new data value *V_pre_*: the previously stored data value
Δ: the relative difference1:begin2: for each (CH[m])3:  transmit *V_new_* to the BS;4:   for each (*V_new_* received by BS from CH[m])5:     Compute Δ;6:        if (Δ < TH)7:          mark cluster [m] as deactivated;8:          send broadcast message (CH[m]_ID, mode_bit = 1);9:         else10:           mark CH[m] as activated;11:        end if12:    end for13:    if (CH[m] received broadcast message from the BS)14:         CH[m] broadcasts a sleep message to its Ns;15:         Ns enter sleep mode;16:         remain CH[m] active;17:       else18:         remain Ns active;19:     end if20: end for

##### Dynamic Sleeping Scenario

In this scenario, the WSN is used in applications that continuously require updates about the surrounding environment. Hence, it is essential for the proposed protocol to adapt to the expected environmental changes throughout the rounds. Furthermore, the proposed protocol assumes that each round consists of a fixed number of identical cycles. When the BS receives the aggregated data sent by the CHs during the initial routing sub-phase, it calculates Δ and compares it with the TH. If Δ is less than the TH, it indicates that the aggregated data lack significant information or do not reflect any noticeable changes. Consequently, the BS sets the mode bit to 1 (deactivated) and broadcasts messages to these CHs, instructing their corresponding clusters to enter sleep mode. In response, the CHs that receive such broadcast messages also notify their associated Ns to switch to sleep mode.

Unlike the static scenario, the proposed protocol assumes that clusters in sleep mode do not remain inactive for the remainder of the round. From this perspective, the remainder of the round is also divided into cycles. A dynamic mechanism, proposed in this section, ensures that the deactivated clusters can wake up again during these cycles.

The active clusters in the first cycle continue sensing the surrounding environment. The dynamic routing approach, as discussed in Section 3.2.4 (Intra-cluster Routing), is employed to forward the sensed data across the two levels toward the corresponding CH. The aggregated data from each cluster are then transmitted toward the BS. The inter-cluster transmission is also performed dynamically using the approach outlined in Section 3.2.4 (Inter-cluster Routing). By the end of the cycle, the BS has a clear understanding of the state of each cluster for the upcoming cycle. In other words, it determines which clusters should remain active, which should be reactivated, and which should be deactivated. At the beginning of each cycle, the BS periodically broadcasts control messages to activate the required clusters and deactivate those that are unnecessary for the current cycle. These decisions are made based on the data received from the currently active clusters and INs located near the sleeping clusters. This operation is repeated in every cycle.

**Figure 7:**
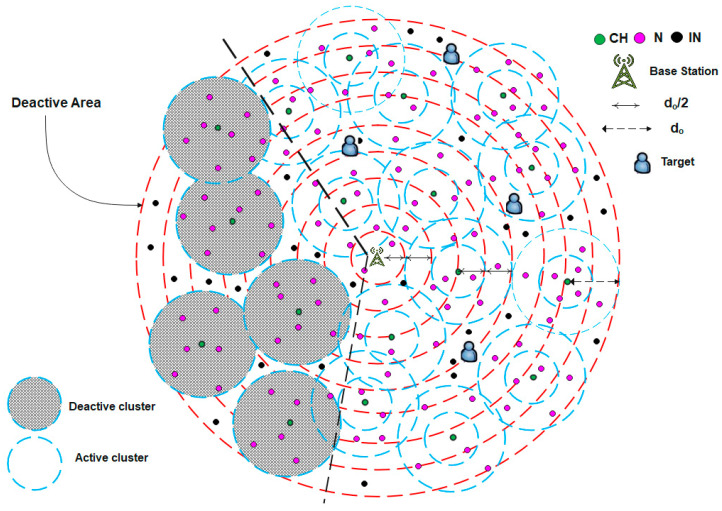
A case example of the dynamic sleeping scenario.

**Figure 8:**
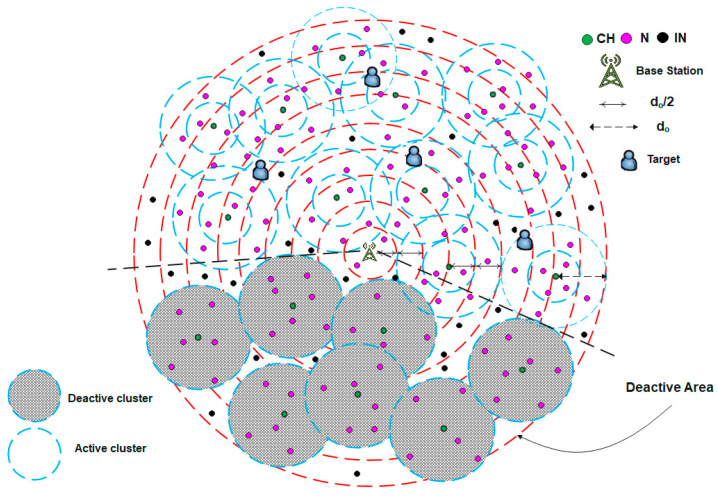
Updated case of the dynamic sleeping scenario shown in Figure 7.

For example, the use of WSNs in target tracking applications requires continuous updates on the coordinates of mobile targets moving within a specific area. The clusters near the target’s position remain active and continue sensing its coordinates, whereas those farther away enter sleep mode, as illustrated in Figure 7. When a target begins to move, as shown in Figure 8, the BS activates the clusters in its new vicinity. This approach provides an efficient method for detecting the target’s location, reducing energy consumption, and prolonging the network lifetime. Algorithm 4 illustrates the pseudocode corresponding to this scenario.
**Algorithm 4**: Dynamic Sleeping Scenario
**Definitions**
CH[m] ∈ the set of CHs

TH: Threshold value N: Normal node
*V_new_*: the new data value *V_pre_*: the previously stored data value
Δ: the relative difference
Max_Cycle: maximum cycle number in the round1:begin2:// cycle 1: Initial Evaluation3:  for each (CH[m])4:    transmit *V_new_* to the BS;5:    for each (*V_new_* received from the BS)6:      compute Δ;7:       if (Δ < TH)8:          mark cluster [m] as deactivated;9:          send broadcast message (CH[m]_ID, mode_bit = 1);10:        else11:         mark CH[m] as activated;12:       end if13:    end For14:         If (CH[m] received a broadcast message from the BS)15:             CH[m] broadcasts a sleep message to its Ns;16:             Ns of this cluster enter sleep mode;17:             remain CH[m] active;18:           else20:             remain Ns active21:         end if22: end for23:// from cycle 2 to the maximum number of cycles in the round24: while (cycle ≤ Max_cycle)25:   for each (CH[m])26:    if (CH[m] received reactivate message)27:       CH[m] broadcasts activate message to its Ns;28:       Ns of the cluster [m] start sensing and transmit their data to CH[m];29:     else30:       the cluster[m] remains in deactivate mode;31:    end if32:     if (CH[m] received a deactivate message from the BS)33:       CH[m] broadcasts a sleep message to its Ns;34:       Ns of this cluster enter sleep mode;35:       remain CH[m] active;36:     end if37: cycle++;

## 5. Simulation and Results

**Figure 9:**
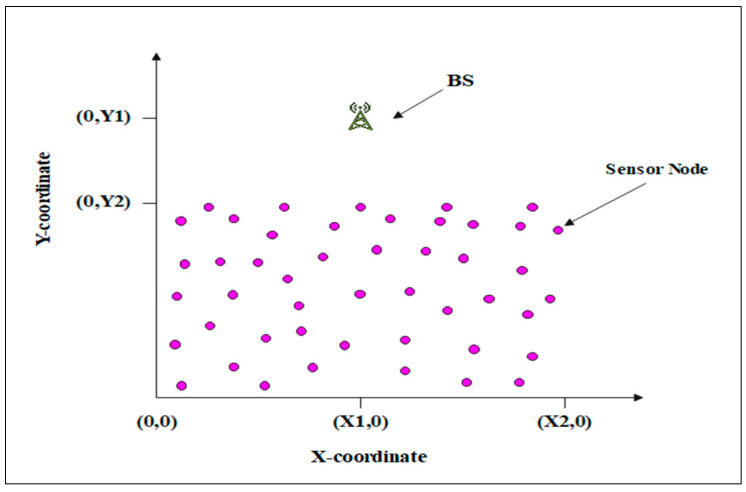
The experimental deployment region.

In this section, the performance of the proposed protocol is evaluated in both of its aspects: the static scenario (EDMR-S) and the dynamic scenario (EDMR-D). The experimental results are compared with those achieved using the DMR protocol. The comparison is further extended to include the EDMHT-LEACH and the LEACH protocols. To verify the effectiveness of the proposed protocol, a number of experiments were performed using MATLAB R2023a. These experiments were conducted in different deployment areas to simulate various scenarios typically faced in WSN environments. The sensor nodes were stationary and randomly distributed across the deployment region, whereas the BS was placed outside this area (Figure 9). In the first experiment, 200 sensor nodes were randomly scattered across three deployment areas, while the BS was positioned at the coordinates (150, 480). Table 2 contains a list of common parameters used in the assessment of these protocols, whereas the elementary components of the first experiment are presented in Table 3.

**Figure 10:**
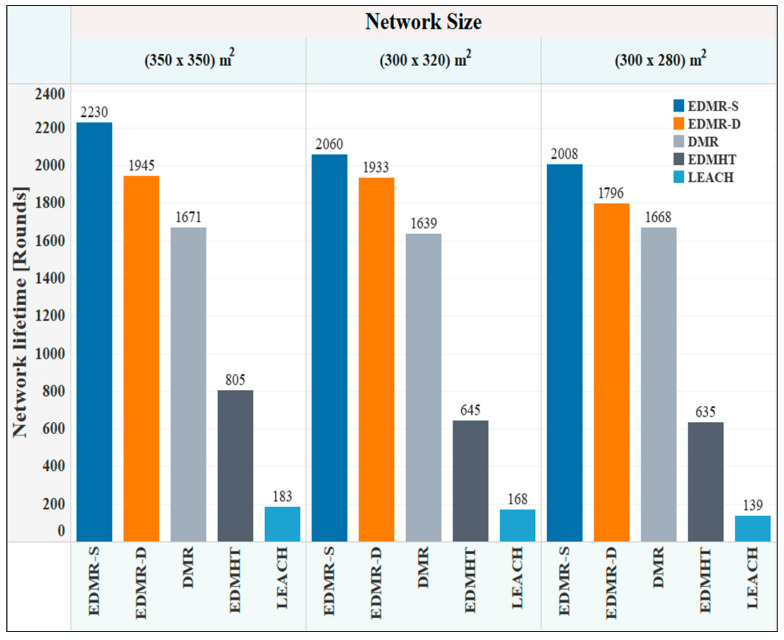
Network lifetime vs. network size for the EDMR-S, EDMR-D, DMR, EDMHT, and LEACH protocols.

Figure 10 demonstrates the network lifetime, measured in rounds, of the EDMR-S and the EDMR-D protocols compared with the other protocols. Generally, the simulation results showed that the network lifetime was extended across all deployment areas through the use of the proposed protocols. For example, in the (350 × 350) m^2^ deployment area, the EDMR-S protocol attained a maximum number of rounds (2230 rounds), while the EDMR-D protocol achieved 1945 rounds. In comparison, the DMR protocol reached only 1671 rounds. As a result, the proposed sleeping mechanism led to remarkable results in the network lifetime compared with the DMR protocol. Similar results were observed in the other two deployment areas. Furthermore, it was noted that the EDMR-S protocol showed a relative advantage over the EDMR-D protocol. This advantage is attributed to the static sleeping mechanism applied in the EDMR-S protocol, which effectively reduced energy consumption by deactivating unnecessary clusters for the remainder of the round.

Notably, in the 350 × 350 m^2^ deployment area, the vertical distance between the node deployment region and the BS is shorter than in the other experimental regions. This proximity minimizes the average transmission distance, which in turn lowers energy consumption and contributes to extending the network lifetime.

**Figure 11:**
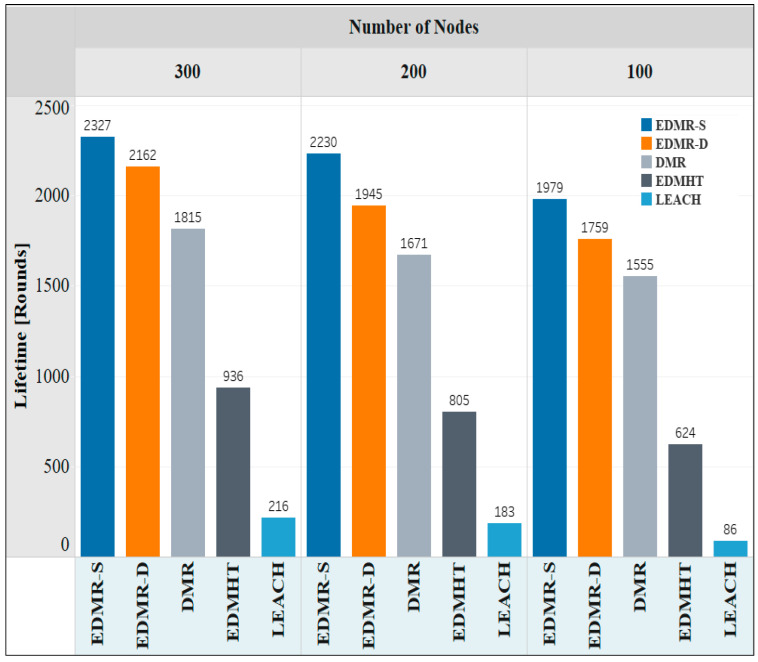
Network lifetime vs. number of nodes for the EDMR-S, EDMR-D, DMR, EDMHT, and LEACH protocols.

The second experiment focused on studying the effect of the number of sensor nodes on the network lifetime. The deployment area (350 × 350) m^2^ was selected; the BS was placed at (150, 480), whereas the number of nodes was varied. Table 4 contains the parameters of the second experiment. Figure 11 shows the impact of varying the number of nodes on the network lifetime. It was observed that the EDMR-S and the EDMR-D protocols outperformed all other protocols for different numbers of nodes used in the experiment. For instance, when 300 sensor nodes were used, the EDMR-S protocol recorded 2327 rounds, the EDMR-D protocol reached 2162 rounds, while the DMR, EDMHT, and LEACH protocols attained 1815, 936, and 236 rounds, respectively. As the number of nodes decreased in the other two experiments (100, and 200 nodes), the overall network lifetime also declined for all protocols. However, the EDMR-S and the EDMR-D protocols continued to surpass the other protocols, even when the number of nodes was reduced.

**Figure 12:**
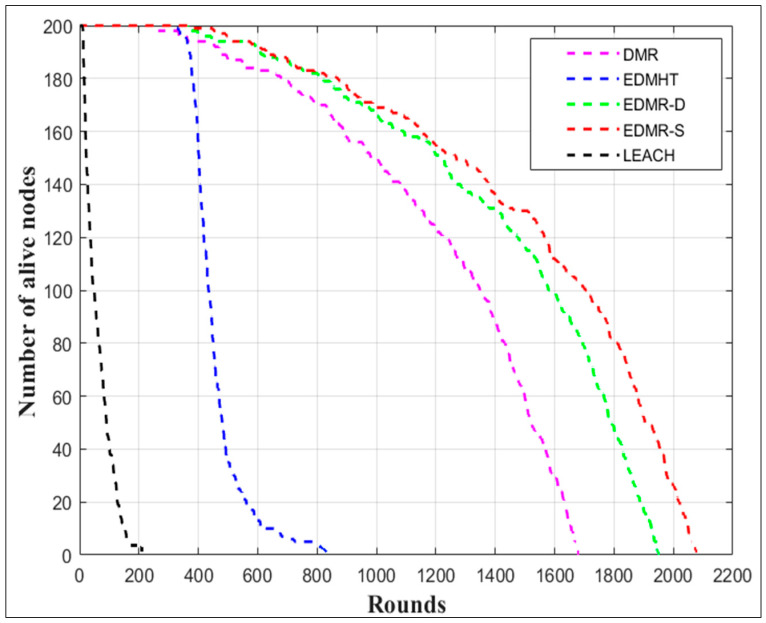
Number of alive nodes vs. number of rounds for the EDMR-S, EDMR-D, DMR, EDMHT, and LEACH protocols.

Figure 12 illustrates the number of alive nodes over the simulation rounds. As is evident, the EDMR-S and the EDMR-D protocols significantly extended the network lifetime compared to the DMR protocol and other evaluated protocols. The proposed protocols and the DMR protocol maintained a moderate decline in the number of alive nodes throughout the rounds. In contrast, the EDMHT and LEACH protocols showed a rapid decline in the number of alive nodes. Notably, the EDMR-S protocol demonstrated relative superiority over the EDMR-D protocol due to its static sleeping mechanism, which keeps the inactive clusters in sleep mode for the remainder of the round.

**Figure 13:**
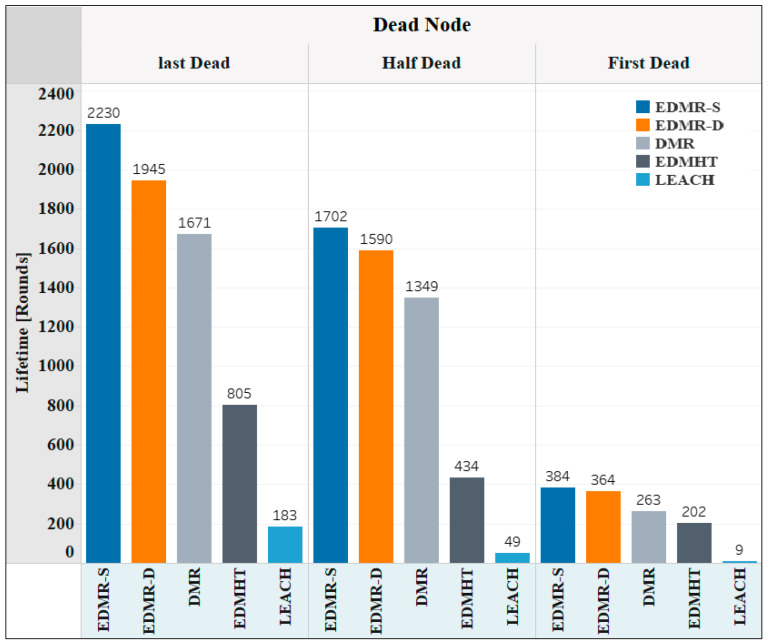
Network stability using the EDMR-S, EDMR-D, DMR, EDMHT, and LEACH protocols.

The stability of the network under all the evaluated protocols is shown in Figure 13. Three main moments were selected: the first dead node, the half-dead node, and the final dead node. As observed, the EDMR-S and the EDMR-D protocols required 384 and 364 rounds, respectively, before the first node died, representing the highest number of rounds among the evaluated protocols. Likewise, the EDMR-S protocol reached 1702 rounds before 50% of the network nodes died, whereas the EDMR-D protocol required 1590 rounds. In comparison, the DMR, EDMHT, and LEACH protocols required 1349, 434, and 49 rounds, respectively. Regarding the final dead node, the proposed protocols once again yielded the highest number of rounds compared to the other protocols. Hence, the proposed protocols enhanced the network stability compared to the DMR protocol.

**Figure 14:**
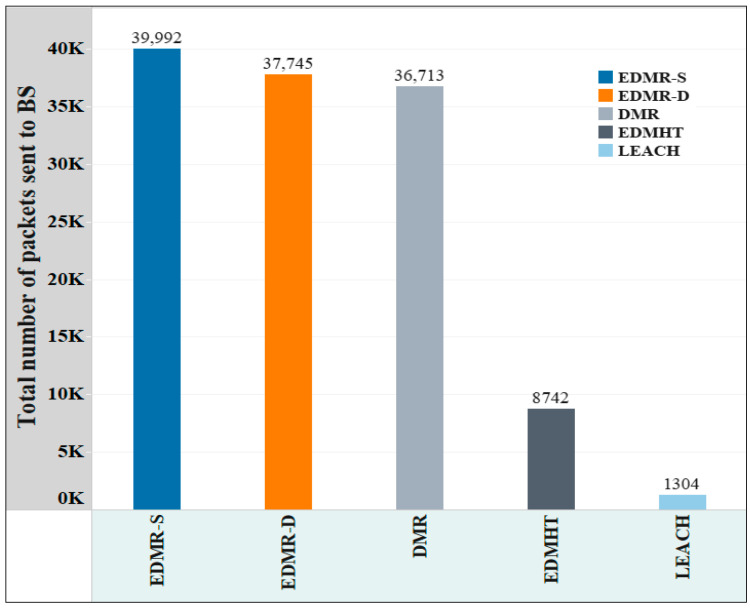
The overall account of packets transmitted to the BS in the EDMR-S, EDMR-D, DMR, EDMHT, and LEACH protocols.

Figure 14 demonstrates the overall number of packets transmitted to the BS for each evaluated protocol. As shown, the EDMR-S and EDMR-D protocols achieved the highest number of packets sent to the BS. This result was obtained because the network lifetime was extended by the proposed protocol compared to the other protocols. Nevertheless, a noticeable similarity was observed in the number of packets sent to the BS between the proposed protocols and the DMR protocol, despite the relative advantage of the proposed approach. Specifically, applying the EDMR-S protocol to the WSN resulted in 39,992 packets being sent to the BS, while the EDMR-D protocol achieved 73,745 packets, compared to 36,713 packets delivered by the DMR protocol. This outcome was attributed to the mechanism used in the proposed protocol, which reduced the number of activated clusters. As a result, the number of packets delivered to the BS was reduced.

## 6. Conclusions

In this paper, the EDMR protocol was proposed to enhance the DMR protocol. It introduces a new sleeping mechanism to reduce the overall energy consumption in WSNs. Clusters that either lack updated data or are located far from the target are deactivated and enter sleep mode. The EDMR protocol assumes that the network lifetime is divided into rounds. Accordingly, each round is divided into two main phases: the network architecture phase and the routing phase.

The EDMR protocol supports two types of applications: monitoring applications and event-driven applications. The EDMR-S protocol is dedicated to monitoring applications, while the EDMR-D protocol is designed for event-driven applications. Both protocols were evaluated against the DMR, EDMHT-LEACH, DMHT-LEACH, and the traditional LEACH protocols. The simulation results revealed that both the EDMR-S and EDMR-D protocols improved the network performance compared to the other protocols, with a relative performance advantage observed for the EDMR-S protocol. Specifically, they prolonged the network lifetime, improved the network stability, and increased the network throughput.

The EDMR protocol may be further extended by incorporating energy-harvesting mechanisms, which enable nodes to recharge their batteries from environmental sources such as solar or thermal energy. Recent research on ambient energy-harvesting in WSNs has proposed various techniques that utilize solar, thermal, and vibration energy resources, achieving considerable improvements in the network lifespan. For instance, the High-Coverage Energy-Harvesting with Uneven Clustering (HCEH-UC) [24] offers an approach to adapt clustering and sleep cycles based on harvested energy levels, which in turn enhances sustainability in long-term deployments. In addition, the EDMR protocol could be evaluated under alternative conditions, including the use of mobile nodes and the initialization of nodes with different initial energy levels.

## Figures and Tables

**Table 1:** Comparative summary of EDMR and related protocols.

Reference	Protocol Name	Sleep Scheduling Mechanism	Sleep Scheduling Control Level	Routing Method	Application-Adapted Sleep Mechanism	Independent Node Unit (IN)
[9]	MH-LEACH	No	-	Multi-hop (Static)	No	No
[10]	LEACH-TL	No	-	Multi-hop (Static)	No	No
[11]	EDMHT	No	-	Multi-hop (Dynamic)	No	Yes
[12]	K-means	Yes	CH-Level	Single-hop	No	No
[13]	EESAA	Yes	Pair-based Level (Pair of Nodes)	Single-hop	No	No
[14]	SEED	Yes	Node-Level	Single-hop	No	No
[15]	T-LEACHSAS	Yes	CH-Level	Single-hop	No	No
[16]	IT2FUSS	Yes	Node-Level (Fuzzy)	Single-hop	No	No
[17]	ETAAR	Yes	CH-Level	Multi-hop (Static)	No	No
[18]	DE-MAC	Yes	CH-Level	Single-hop	No	No
[19]	RMIS	Yes	CH-Level	Single-hop	No	No
[20]	RL-Coop Event	Yes	Cluster-Level	Single-hop (Intra-cluster)	No	No
-	EDMR	Yes	BS-Level (Centralized)	Multi-hop (Dynamic)	Yes Dual-mode (Static/Dynamic)	Yes

**Table 2:** Standard simulation parameters.

Parameters	Values
BS coordinates (X1, *Y*1)	(150 m, 480 m)
Initial energy (*Eo*)	0.5
The percentage of CH (*p*)	0.2
*E_elec_*	50 nJ/bit
*ε* *_fs_*	10 pJ/bit/m^2^
*T_min_*	0.03
Relative weight (*α*)	100
Data aggregated energy (*E_DA_*)	5 nJ/bit
*ε* *_mp_*	0.0013 pJ/bit/m^4^
TH	0.03
Data packet size	6400 bit
Control packet size	200 bit

**Table 3:** Key simulation parameters used in the first experiment.

Total Nodes	Field Dimensions (*X*2 × *Y*2)	BS Position (*X*1, *Y*1)
200	(350 m × 350 m)	(150 m, 480 m)
200	(300 m × 320 m)	(150 m, 480 m)
200	(300 m × 280 m)	(150 m, 480 m)

**Table 4:** Key simulation parameters used in experiment 2.

Total Nodes	Field Dimensions (x × y)	BS Position (x1, y1)
100	(350 m × 350 m)	(150 m, 480 m)
200	(350 m × 350 m)	(150 m, 480 m)
300	(350 m × 350 m)	(150 m, 480 m)

## Data Availability

The data and pseudocode for the phases of the proposed protocols are provided in this paper.
